# Quantitative Assessment of Abiotic Stress on the Main Functional Phytochemicals and Antioxidant Capacity of Wheatgrass at Different Seedling Age

**DOI:** 10.3389/fnut.2021.731555

**Published:** 2021-08-24

**Authors:** Bianling Jiang, Guizhen Gao, Mengting Ruan, Ying Bian, Fuyun Geng, Weiwei Yan, Xuehua Xu, Mengdie Shen, Jiafeng Wang, Ran Chang, Lisheng Xu, Xingtao Zhang, Fan Feng, Qiong Chen

**Affiliations:** School of Biological and Food Engineering, Suzhou University, Suzhou, China

**Keywords:** wheat young leaves, ultraviolet stress, drought stress, salinity stress, functional compounds, comprehensive nutrition ranking

## Abstract

The wheat seedlings of 6 days old were daily subjected to ultraviolet irradiation (irradiating for 5, 10, 20, 40, and 60 min/day, respectively), Polyethylene glycol 6000 (5, 10, 15, 20, 25% in 1/2 Hoagland solution, respectively), and salinity solution (10, 25, 50, 100, 200 mM in 1/2 Hoagland solution, respectively), while the control group (CK) was supplied only with the Hoagland solution. The wheatgrass was harvested regularly seven times and the total soluble polysaccharides, ascorbic acid, chlorophyll, total polyphenol, total triterpene, total flavonoid, and proanthocyanins content were tested. The antioxidant capacity was evaluated through 2,2′-azino-bis (3-ethylbenzthia-zoline-6-sulfonic acid) (ABTS), 2,2-diphenyl-1-picrylhydrazyl (DPPH) scavenging ability, and ferric ion reducing power. Technique for order preference by similarity to ideal solution (TOPSIS) mathematical model was adopted to comprehensively assess the functional phytochemicals of the different treatments. The results showed that the accumulation patterns of phytochemicals under abiotic stress were complex and not always upregulated or downregulated. The antioxidant activity and functional phytochemicals content of wheatgrass were significantly affected by both the stress treatments and seedling age, while the latter affected the chemicals more efficiently. The top five highest functional phytochemicals were observed in the 200 mM NaCl treated group on the 21st and 27th day, 25% PEG treated group on the 24th day, 200 mM NaCl treated group on the 24th day, and the group of 40 min/day ultraviolet exposure on 27th day.

## Introduction

Wheatgrass, the mature shoots of the common wheat plant (*Triticum aestivum*, Poaceae family) which has been considered the most edible grain cereal-grass crop globally (1), is used as herbal medicine and nutraceutical traditionally (2). The consumption of wheatgrass could be traced to as early as ancient Egypt of 5,000 years ago or Mesopotamian Civilization (3). The recent interests in the wheatgrass was boomed immensely by Dr. Ann Wigmore in 1970s who developed wheatgrass juice as a part of her herbal therapeutic nutritional approach and compiled “The Wheatgrass Book” (4). Wheatgrass was demonstrated to have a wide range of health benefits under conditions, such as common cold, astriction, diabetes, kidney swelling, anemia, eczema, (5) thalassemia, and myelodysplastic syndrome (2), and possess antimutation (6), anti-inflammatory, antioxidant, immunoregulation, hemostasis, diuresis, antimicrobial, antiaging, and anti-cancer (e.g., cervical cancer and oral squamous cell carcinoma) properties (7–9).

The therapeutic properties of wheatgrass could be attributed to the rich phytochemical components, such as chlorophyll, ascorbic acid, bioflavonoids, and so on (8, 10), which varied according to the production process and growing environments (11). Similar views were shared that abiotic stress factors could affect the plant bioactive compounds and produce differences in physiological condition and nutritional value (12, 13). Literature has shown that ascorbic acid, β-carotene, carotenoids, functional phytochemicals that include phenolics, flavonoids, and antioxidant activity of leafy vegetables were augmented under abiotic stresses, such as drought (14, 15) and salinity stress (16, 17). In addition, there were reports on the upregulation of ascorbic acid under abiotic stress during rice seed priming (12), but was observed decrease in wheatgrass and soybean plants under drought stress (18). As to triterpene, it was reported to be augmented in some plants (19). In blue light treated einkorn wheatgrass and red light treated emmer wheatgrass, the phenol and flavonoid content was increased (20). Falcinelli et al. (21) revealed that NaCl treatment could markedly increase the total polyphenols content (TPC) and antioxidant activity in wheatgrass. Jaiswal et al. (22) discovered that selenium and ultraviolet-B radiation or their combination could enhance the flavonoid and phenolic content. Benincasa et al. (23) demonstrated that optimal combination of temperature and time, light modulation, and salt stress would upregulate the phytonutrients such as ascorbic acid, tocopherol, β-carotene, phenols, and flavonoids in some extent. Hence, it was presumed that the plants as refer to wheatgrass would generally produce some phytochemicals, as secondary metabolites, whose role was to help plants cope with the unfavorable environmental conditions. However, all the existing data referring to the abiotic stress effect on phytochemicals of wheatgrass, to our best knowledge, were not integrated and focused mostly on a few kinds of compounds, such as flavonoids, phenols, tocopherol, or carotenes. The previous reported cereal grasses were normally germinated 6–10 days and no more than 14 days. The information that the long-term (exceeding 14 days) accumulation of phytonutrients, such as triterpene, proanthocyanin, soluble polysaccharides, ascorbic acid, and the like under different stress density still remains scarce.

Therefore, to reveal the effect of abiotic stress on the main functional phytochemicals and antioxidant activity, the wheat seedlings were suffered from the different densities of NaCl, PEG, and ultraviolet-C radiation and tested for the ascorbic acid content (AAC), TPC, total triterpenes content (TTC), total flavonoids content (TFC), total proanthocyanins content (TPAC), and total chlorophyll content (TChl) content and the antioxidant activity through 2,2'-azino-bis (3-ethylbenzthia- zoline-6-sulfonic acid) (ABTS) and 2,2-diphenyl-1-picrylhydrazyl (DPPH) assays and ferric ion reducing antioxidant power (FRAP) of different seedling ages.

## Materials and Methods

### Material and Reagents

The wheat seeds were kindly provided by a local farmer in Lingbi County, Suzhou City, Anhui Province, China. Hoagland Nutrition reagent was purchased from Qingdao Hope Bio-Technology Co., LTD (Qingdao, China). Polyethylene glycol 6000 was bought from Wuxi Yatai Allied Chemicals Co., Ltd. Glucose was from Shanghai Zhanyun Chemical Co., Ltd (Shanghai, China). Vanillin was from Sinopharm Chemical Reagent Co., Ltd (Shanghai, China). Ursolic acid, Folin–Ciocalteu's phenol reagent, gallic acid, rutin hydrate, (+)-catechin, L-ascorbic acid, ABTS, DPPH were purchased from Shanghai Macklin Biochemical Co., Ltd (Shanghai, China). (±)-6-Hydroxy 2,5,7,8-tetramethylchromane-2-Carboxylic acid (Trolox) was from Shanghai Aladdin Biochemical Technology Co., Ltd (Shanghai, China). All the reagents were of analytical grade.

### Plant Materials and Abiotic Stress Treatment

#### Abiotic Treatment

The cultivation of the wheatgrass was executed based on the procedures proposed by the team (24). Briefly, 55 g of the thrice-washed seeds were sown evenly in a hydroponic tray sized 32.5 × 24.5 × 4.5 cm in four rectangular pieces, then were semi-immersed in deionized water for 24 h away from light to let malt. The wheat malts were supplied with 1/2 Hoagland solution and cultivated at 22 ± 1°C under 16 h-photoperiod (20 W) light irradiance. The aged 6 days seedlings were exposed to UV-C irradiation daily (40 W, Ozone free, for 5, 10, 20, 40, and 60 min correspondingly noted as UV 5, UV 10, UV 20, UV 40, and UV 60), Polyethylene glycol 6000 (5, 10, 15, 20, and 25% in 1/2 Hoagland solution correspondingly noted as PEG 5%, PEG 10%, PEG 15%, PEG 20%, and PEG 25%), and salinity solution (10, 25, 50, 100, and 200 mM in 1/2 Hoagland solution correspondingly noted as NaCl 10, NaCl 25, NaCl 50, NaCl 100, and NaCl 200), respectively, while the control group (CK) was supplied only with the Hoagland solution. All the media solutions used during the cultivation were 450 ml per tray and refreshed every 2 days. The deionized water was replenished properly on the interval of the culture matrix refreshing to restore the initial weight and keep the matrix concentration constant. The grass was sampled on 9th, 12th, 15th, 19th, 21st, 24th, and 27th day after seeding (noted as D9, D12, D15, D21, D24, and D27, respectively) and immediately frozen in liquid nitrogen (LN_2_) and stored at −80°C until use.

#### Preparation of Wheatgrass Extracts

Grass extracts to be analyzed for TTC, TPC, TFC, TPAC, and antioxidant capacity were prepared as followings: the wheatgrass was well ground in LN_2_ and 1.0 g sample of each treated group were ultrasonic extracted thrice (80 W, 20 min) with 25 ml of 80% methanol. The three filtrates were pooled and brought to 100 ml. The extracts for total soluble polysaccharides content (TSPC) analysis were prepared as: the above pellets were again aqueously extracted thrice in boiling water-bath for 30 min and standardized to 100 ml. The methanol extracts were stored at −20°C until use while the aqueous extracts were stored at 4°C and detected within 3 days. In the preparation, 112 samples in total were yielded and tested for the seven kinds of compounds and three antioxidant activity indices. All the tests were applied in a microplate reader (Thermo Fisher Scientific, Type 1510).

### Determination of TTC

The TTC was measured using the methods described by Siyuan Luo et al. (25) with slight modifications. In this method, 100 μl extracts or ursolic acid standard solutions (20–120 μg ml^−1^) were mixed with 100 μl vanillin-acetic acid (2.5%) and 200 μl perchloric acid. After 60°C incubation for 15 min, 650 μl glacial acetic acid was added and well-mixed. In addition, 300 μl of the mixture was pipetted to a 96-well-microplate (CELLSTAR® from Greiner Bio-One GmbH) and read at 550 nm using the microplate reader after standing for 10 min. The TTC was calculated from the standard curve and expressed as milligram ursolic acid equivalents/gram (mg UAE/g) of fresh weight (FW).

### Determination of TPC

The determination of TPC was performed using the Folin–Ciocalteau method previously described by Sarker and Oba (26). The extracts (100 μl) or series of standards (12.5, 25, 50, 100, 150, and 200 μg ml^−1^ gallic acid) were added. After reagent mixing and reaction, 300 μl was moved to a 96-well-plate and read at 740 nm. The results were estimated as equivalent to gallic acid standard (mg GAE/g FW).

### Determination of TFC

The TFC was determined according to an assay described by Guo et al. (27). Briefly, the addition volume of the extracts or standard rutin solution (20–100 μg ml^−1^), NaNO_2_ solution (5%, w/v), Al (NO_3_)_3_ solution (10%, w/v), and NaOH solution (1 mol/L) were adjusted to 2.0, 0.2, 0.2, and 2.0 ml, respectively. The mixture (300 μl) was pipetted to a 96-well-plate and read at 510 nm. The amount of TFC was expressed as rutin equivalents/gram (mg RE/g FW).

### Determination of TPAC

The TPAC was detected by using a method previously reported by Zribi et al. (28) which was adjusted to be feasible in the microplate reader. The volume of extracts or standard catechin solution (4–32 μg ml^−1^), 4% vanillin–methanol (w/v), and hydrochloric acid were adjusted to 200 μl, 1.0 ml, and 0.5 ml, respectively. The mixture (300 μl) was transferred to a 96-well-plate and read at 500 nm. TPAC was presented as milligram catechin equivalent/gram fresh weight (mg CE/g FW) using the catechin calibration curve.

### Determination of TSPC

The TSPC was detected based on phenol-sulfuric acid assay described by Lei Guo et al. (29), of which the reagent volume was diminished aliquot for high throughput detection. Briefly, 0.2 ml of aqueous extracts or standard glucose solution (10–60 μg ml^−1^) was mixed with 0.2 ml 5% distilled phenol solution and 1 ml concentrated sulfuric acid. After vortex and 30 min standing at room temperature, the mixture (300 μl) was transferred and read at 490 nm. The results were obtained using a calibration curve from standard glucose solutions and expressed as milligram glucose equivalent/gram in fresh weight (mg GE/g FW).

### Determination of Chlorophyll Content

The chlorophyll (such as chlorophyll a noted as Chl *a*, chlorophyll b noted as Chl *b*, and total chlorophyll noted as TChl) was extracted based on the procedure reported by Mashabela et al. (30) with modifications and detected by using the methods previously reported by Warren (31). Specifically, the wheatgrass samples were well-ground in liquid nitrogen and 1.0 g was weighed and macerated in 25 ml methanol at room temperature for 48 h. Afterward, the resulting mixtures were passed through a 0.22-μm nylon filter and diluted 1-fold. The dilutions of 200 μl were removed to a flat-bottomed 96-well-plate and read at 652 and 665 nm. The chlorophyll concentration was calculated from the following formula:

Chl *a* (μg mL^−1^) = −8.0962 A_652, 1cm_ + 16.5169 A_665, 1cm_

Chl *b* (μg mL^−1^) = 27.4405 A_652, 1cm_ – 12.1688 A_665, 1cm_

TChl = Chl *a* + Chl *b*

A_652, 1cm_ = (A_652, microplate_ - blank)/0.51

A_665, 1cm_ = (A_665, microplate_ - blank)/0.51

Where, A_652, 1cm_ and A_652, microplate_ represents the absorbance at 652 nm in a spectrophotometer and a microplate reader, respectively; A_665, 1cm_ and A_665, microplate_ represents the absorbance at 665 nm in a spectrophotometer and a microplate reader, respectively.

The results were expressed as milligram/gram fresh weight (mg/g FW).

### Determination of AAC

The AAC was determined by a second-order derivative spectrometer method (32, 33). The wheatgrass samples of 2.0 g were ground and macerated in 100 ml 1.0 M HCl which was gradually added for 10 min. The extracts were passed through a 0.45-μm aqueous syringe filter and quantitatively diluted 5-fold. The peak-baseline amplitudes of the filtrates in the second-order derivation absorption spectra at 267.5 nm (detected using Type U-3900 UV/VIS spectrometer from HITACHI High-Tech Science Corporation, Tokyo, Japan) was used to construct the calibration curve of standard ascorbic acid solutions with the concentration ranging from 5 to 30 μg ml^−1^. The results were expressed as milligram ascorbic acid/g of FW.

### Antioxidant Activity

#### ABTS Radical Scavenging Assay

The detection of ABTS bleaching ability was conducted using the method described by Lin et al. (34) with slight adjustments. An equal volume (25 ml) of 7.4 mM ABTS solution and 2.6 mM aqueous K_2_S_2_O_8_ were mixed at room temperature for at least 16 h away from light to generate ABTS•^+^ stock solution. Afterward, the stock solution was diluted using 5 mM pH 7.4 PBS to the absorbance of 0.75 at 734 nm in a 96-well-plate to prepare the working solution, of which 200 μl was mixed with 40 μl of the extracts or standard Trolox solution (0.02–0.16 mM). After incubation for 6 min in the dark and vibration for 15 s, the absorbance was read. The results were calculated from the Trolox calibration curve and expressed as milligram Trolox Equivalents/gram FW (mg TE/g FW).

#### DPPH Radical Scavenging Assay

The DPPH radical scavenging ability was evaluated according to a previously described method (34) with minor adjustment. The solution (200 μl) of 0.15 mM DPPH was blended with 100 μl of the extracts or standard Trolox solution (0.02–0.16 mM) in a 96-well-plate. After 37°C incubation for 30 min and 15 s vibration, the absorbance was read at 517 nm vs. 80% methanol blank. The results were expressed as milligram TE/gram FW.

#### FRAP Assay

The FRAP assay was conducted by using the method described by Lin et al. (34).

### TOPSIS Model Establishments

The TOPSIS model proposed by Hwang and Yoon (35) was used to evaluate the comprehensive nutrition value of all the treated groups. The decision matrix was established as X = *(X*_*ij*_*)*_*m×n*_, where m means the different abiotic groups, such as five treatments of PEG, five treatments of NaCl, five treatments of ultraviolet, and a CK, while n represents seven criteria that include TTC, TPC, TFC, TPAC, TSPC, TChl, and AAC. The weight of individual criterion ω_i_ was 1.0, 1.0, 1.0, 0.5, 1, 1.5, and 1.0, respectively.

### Statistics Handling

All the tests were performed in triplicates and the results were shown as mean ± SD. Duncan's new multiple range was used for the difference analysis tests. Correlation-ship was analyzed using Pearson's correlation. A multi-factor analysis of variance (MANOVA) was conducted to examine which factor (seedling age or various abiotic treatments) dominates the corresponding phytochemicals. The principal component analysis (PCA) was also carried out. All the statistics handling were carried out using SPSS software (IBM Corp. USA, version 20.0). Asterisks indicated significant differences (^**^p < *0.01*, ^*^p < *0.05*). The heatmap hierarchical clustering analysis (HHCA) of the measured functional compounds was run using TBtools software.

## Results and Discussion

### Triterpene

**Figure 1:**
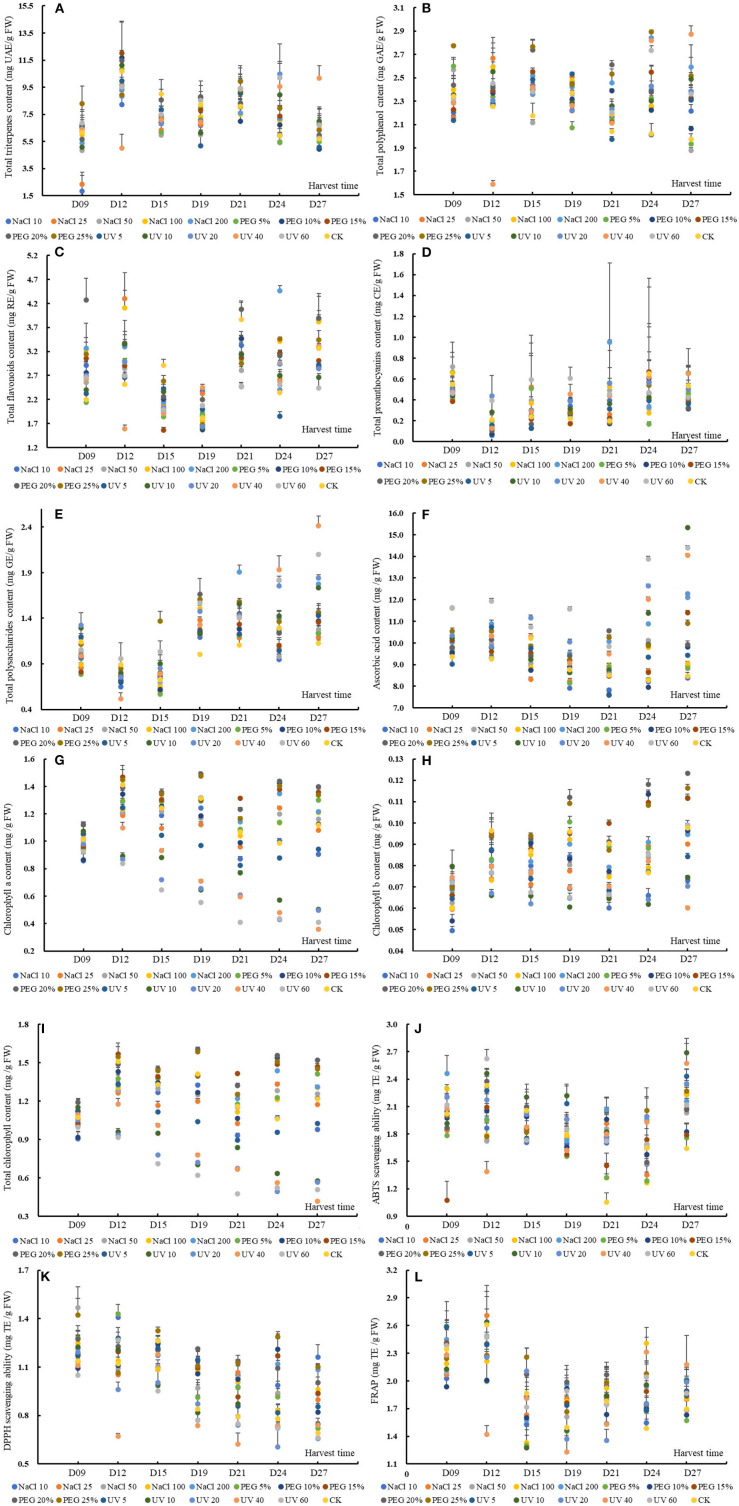
The content or value of total triterpenes **(A)**, total polyphenols **(B)**, total flavonoids **(C)**, total proanthocyanins **(D)**, total soluble polysaccharides **(E)**, ascorbic acid **(F)**, chlorophyll a **(G)**, chlorophyll b **(H)** and total chlorophyll **(I)**, 2,2'-azino-bis (3-ethylbenzthia- zoline-6-sulfonic acid) (ABTS) **(J)**, and 2,2-diphenyl-1-picrylhydrazyl (DPPH) **(K)** scavenging ability and ferric iron-reducing antioxidant power **(L)** of wheatgrass on the 9th~27th day after seed sowing. A total of 112 samples (16 groups × 7 times of harvesting) were analyzed. The results were expressed as mean ± SD.

The TTC of wheatgrass ranged from 1.83 ± 1.40 to 12.00 ± 2.30 mg UAE/g FW, shown in Figure 1A. The results of MANOVA showed that the TTC was significantly affected by abiotic treatment and seedling age, while seedling age determined dominantly the TTC other than abiotic treatment [*p* < 0.01, *F*_(seedlingage)_ = 76.02 > *F*_(abiotictreatment)_ = 5.24]. Previous data indicated that UV-B treatment could contribute to an increment of TTC in the *Adhatoda vasica* plant (36) and that water deficit could stimulate the triterpene accumulation in *Hypnum plumaeforme* (but not in *Pogonatum cirratum*) (37) or that abiotic stress-induced the accumulation of some triterpenes in *Quillaja brasiliensis* (38). However, in the present study, based on MANOVA, we found that the NaCl 10, NaCl 25, and NaCl 50 groups had a lower TTC than the CK which showed no significant differences between the other groups like UV-, PEG-, or NaCl 100 and NaCl 200- treated group (*p* < 0.05). As a kind of UV-absorbing secondary metabolites, the accumulation content of triterpene was impacted by the impairment of metabolism and the upregulation of relative genes and enzymes (36, 38). The results suggested that UV exposure affected the triterpene minorly before D24 compared to control might be explained by the balance of the impairment and upregulation or by the different interactions of individual triterpene that existed in wheatgrass because there was a report that revealed some triterpene compounds were not affected by UV (36). On D24 and D27, that the TTC was upregulated non-/significantly in UV 10–60 groups (compared with control) might be due to the loss of water. The reason that the TTC was not incremented by PEG and salt stress could be as well-attributed to the different reactions of individual triterpene contained in wheatgrass. It might be inferred that the triterpene upregulation was not the pathway for wheat to cope with unfavorable conditions.

An obvious accumulation peak was noticed on D12 and D21 (based on MANOVA, *p* < 0.05), which shared a similar changing pattern with the previous study in UV-treated barley grass where the peak appeared on D15 and D21 (24). Specifically, except for UV40 and PEG 25% dose group, the TTC of other groups increased significantly on D12. The group of NaCl 10, NaCl 50, PEG 25%, UV5, UV10, and UV20 had a second peak TTC on D21. The TTC of wheatgrass could rival some fruit, such as Chinese jujube (TTC ranged from 7.52 ~ 16.57 mg UAE/g FW) (39).

### Polyphenol

The TPC of wheatgrass in the present study varied from 1.59 ± 0.03 to 2.89 ± 0.01 mg GAE/g FW (Figure 1B). The MANOVA results showed that both the abiotic treatments and seedling age could affect the TPC significantly while the former worked more effectively [*p* < 0.01, *F*_(seedlingage)_ = 6.36 > *F*_(abiotictreatment)_ = 5.14]. Generally, except for the treatments of 50 mM NaCl, 5% PEG, 10% PEG, and 5-min-UV, the other treatments could induce a higher TPC than the control. The accumulation patterns of the TPC of all the groups were presented in a wavy manner and peak values appeared on D15 and D24 based on MANOVA. The TPC of the results from D9 to D12 was consistent with a previous study that no significant differences during quinoa malting were found when the seeds were treated with different wavelength light or solutions (40). Specifically, on D15 all the groups except NaCl 50 had a higher TPC than the control. On D21 and D24 except for group UV5, and on D27 except for group PEG 5% and PEG 10%, all the other groups showed higher TPC than the control, suggesting that abiotic treatment could improve or reserved the TPC under prolonged treating time. Differentiated results were found on whether the TPC increased or declined by abiotic stress (16, 40–42). Hence, it was explained that TPC generally stayed stable from D9 to D12 when the wheatgrass was struggling to adapt itself to the adverse conditions, then incremented to handle the situation. The different reactions of TPC between the previous reports and the results attributed to cultivar variations.

The TPC was much higher than some vegetables, such as sweet potatoes (43), or leafy vegetables such as amaranth (44, 45), and common fruits, e.g., apple, apricot, cherry, peach, plum (46), banana, mango, papaya, passion fruit, and so on (47).

### Flavonoid

The TFC of wheatgrass in the present study ranged from 1.56 ± 0.05 to 4.47 ± 0.11 mg RE/g FW, shown in Figure 1C. In the present study, different intensity of UV stress or PEG-simulated drought was probed on the accumulations of flavonoid and found that the TFC were prone to be more susceptible to the seedling age rather than these abiotic treatments (including NaCl stress), on which the flavonoid value upregulated or not depended [*p* < 0.01, *F*_(seedlingage)_ = 71.06 > *F*_(abiotictreatment)_ = 10.77, data from MANOVA results]. The overall accumulation patterns of the total flavonoid varied significantly with the seedling age and three accumulation peaks appeared, respectively, on D12, D21, and D27. The MANOVA results showed that the 25 mM NaCl -, 100 mM NaCl -, 200 mM NaCl -, and 20% PEG treatment could improve the TFC. The previous studies reported that the flavonoid could be enhanced by drought treatment within 48 h in wheat leaves (48) or by violet treatment for 30 days in root culture of *Setiva rebaudiana* (49). It has been postulated that the TFC could be enhanced by abiotic stress, such as drought, salinity, and UV stress (41), however, in the results, the situation was complicated. Specifically, all the abiotic treated groups (except for PEG 5% on D9, UV40 on D12, and UV5 on D24) had non-/significantly higher TFC than the control on D9, D12, and D24. In contrast, all the treated groups (except for NaCl 100 on D21) showed a significantly lower TFC content than the control on D15 and D21. The accumulation patterns of TFC varied with the difference of treatments intensity, which could be explained by that the increase or decrease of some secondary metabolites in plants depends on the sensitivity of the plants to this type of stress condition (50). The reason for the fluctuation of TFC could be attributed to the genetic regulation mode of these compounds.

The TFC in the present study was much higher than some green vegetables, such as amaranth (41, 51), lettuce, salad spinach, mitsuba, pok choi, mizuna, komatsuna in Gifu (Japan) (52) and was in the same level of some Thai fruits, such as egg fruit, manila tamarind, otatheite apple, ivy gourd, and governor's plum (53) or tomato and lotus root (54, 55).

### Proanthocyanin

The TPAC, ranging from 0.06 ± 0.02 to 0.95 ± 0.76 mg CE/g FW (Figure 1D), was not a remarkable functional phytochemical in wheatgrass but was still fell into the same level with rice of Sri Lanka (56), or some fruit, such as jujube (39), pomegranate (57), or even wild berries from Himalaya area (58). The overall TPAC changing patterns were found to decrease remarkably from D9 to D12 and then uprose gradually (*p* < 0.05) on basis of MANOVA. The previous studies revealed that condensed tannins (proanthocyanins) were ubiquitous in ligneous pants but almost absent in herbaceous species (59), which was in accordance with this study results. It was known from the MANOVA results that the seedling age significantly affected the TPAC (*p* < 0.05) while abiotic treatments did the little effect on the content. Saoussen et al. (60) found that salt stress could significantly increase the TPAC content of Tunisian safflower (a medicinal plant), however, in the present study results, no significant differences were noticed between the CKs and stress treatment groups (*p* < 0.05). To address specifically, UV20 and UV40 on D12, UV5, UV20, UV40, and UV60 on D19, and NaCl 100 on D21 had remarkably higher TPAC than the control. An interesting finding was that the higher TPAC compared to the control was mostly observed in UV treated groups, suggesting that certain intensity of UV stress could promote the proanthocyanins synthesis in some particular seedling age despite of the genetic restriction of this species.

### Soluble Polysaccharide

The TSPC of wheatgrass in the present study ranged from 0.52 ± 0.07 to 2.41 ± 0.11 mg GE/g FW (Figure 1E). Some previous studies showed that the TSPC decreased significantly with the ripening or seedling age (24, 61). In this study, all the tested groups shared similar accumulation patterns of TSPC that decreased from D9 to D12 and then climbed up significantly (*p* < 0.05). Based on the MANOVA results, the TSPC was significantly affected by the seedling age and abiotic treatments, among which the seedling age influenced the content more efficiently [*p* < 0.01, *F*_(seedlingage)_ = 136.80 > *F*_(abiotictreatment)_ = 13.84]. Only 200 mM NaCl-, 20% PEG-, 25% PEG-, and UV-treatment (in exception of UV5) could enhance the TSP content (*p* < 0.05) and these results were subtly diverse with previous reports that salinity exposure enhanced the TSPC during germination and seedling period (62) or PEG limited low-molecular-mass TSPC (63). From D9 to D12, most of the abiotic groups (except UV20 and UV60) showed a lower TSPC than the control. When it was on D19, D21, and D27, all the treated groups showed a higher TSPC than the control. It could be explained that the decreased TSP from D9 to D12 was converted to monosaccharides and the latterly increased TSP was from the breakdown of wheat cell wall under the stress conditions (64), which was indirectly validated by previous reports that the activity of relative glycoside-hydrolyzing enzymes was increased under drought stress (64).

Besides, the TSPC in the current study was found to be much lower than some common fruits and vegetables (65) but still rival to medicinal-use fruit like jujube cv. *Dazao*, jujube cv. *Junzao*, and jujube cv. *Huizao* (66).

### Ascorbic Acid

The AAC of the wheatgrass in the present study was ranging from 7.59 ± 0.10 to 15.32 ± 0.05 mg/g FW (Figure 1F). Based on MANOVA results, the AAC could be significantly affected by the seedling age and abiotic treatments [*F*_(seedlingage)_ = 25.39 > *F*_(abiotictreatment)_ = 23.86; *p* < 0.01]. The overall accumulation patterns of AAC gradually were observed to be decreased from D9 to D21, then increased from D21 to D27. Through MANOVA, 200 mM NaCl-, 20% PEG-, 25% PEG-, and UV-treatment (in exception of UV5) could significantly enhance the AAC (*p* < 0.05). An interesting founding was that the comparative higher AAC than the control was observed in intense abiotic stress groups, which was discording with previous studies that AAC declined under salt stress (30–40 days) in all the tested wheat genotypes and the decreasing magnitude augmented with salinity levels (67) and that drought decreased the AAC (68). There was a literature showing a similar result with ours that UV radiation treatment for 2 weeks elevated the AAC in Arabidopsis thaliana (69).

The different abiotic treatments showed different accumulation modes. More specifically, from D9 to D12, all the groups had a higher AAC than the control excluding UV5 on D9. In addition, on D24 and D27, most of the abiotic groups showed a higher AAC than the control except PEG 10% and NaCl 10 on D24 and NaCl 10/50 on D27. However, on D15, except groups of UV20–UV60, all other abiotic treatments induced a significantly lower AAC than the control. The literature with respect to the effects of abiotic stress on AAC mostly was of single-point sampling or no more than three times sampling throughout stressing period and concluded if the AAC was elevated or suppressed. However, the AAC response to stressors was regulated complicatedly by a series of successive biochemical reactions, activation or inhibition of relative enzymes, synthesis of other protective substances, and so on (70), hence, it was vitally important to take the growth time and stress intensity as well as cultivars into consideration.

The AAC in the results was much higher than most of the common fruits known as their high ascorbic acid value, such as strawberry, lemon, orange, kiwifruit, mandarin, mango (71), and leafy vegetables, such as amaranth (72, 73).

### Chlorophyll

The Chl a content varied from 0.36 ± 0.00 to 1.49 ± 0.01 mg/g FW (Figure 1G), which was overwhelmingly higher than the Chl b content (0.049 ± 0.002 to 0.123 ± 0.001 mg/g FW, Figure 1H), which conforms with the results of leafy vegetable amaranth (74). The TChl content ranged from 0.42 ± 0.00 to 1.60 ± 0.02 mg/g FW (as shown in Figure 1I). The TChl content was more significantly affected by the different abiotic treatments [*F*_(seedlingage)_ = 21.56 < *F*_(abiotictreatment)_ = 62.12, *p* < 0.01] than the seedling age. PEG the 10 mM NaCl and UV treatments significantly decreased the chlorophyll content while 15~25% treatments could markedly upregulate it compared to control (*p* < 0.05). PEG was usually used to simulate the drought stress. There are reports that the drought stress decreased the chlorophyll or have no significant effect on chlorophyll concentration and that the chlorophyll was increased in some drought-tolerant wheat cultivars after 39 days of stress (75). The enhancement of TChl in young wheat leaves in the present study could be due to the activation of the enzyme in the light-dependent stage of biosynthesis (76). The TChl content was decreased non-/significantly under salinity stress from D9 to D21 but was increased on D24 compared to control. A previous study revealed that some wheat cultivars recorded a higher TChl at the tittering stage (77), which shared roughly consistent with the results to some extent. It could be inferred that the salinity stress could increment the TChl at some particular stage and UV stress would promote the degradation of chlorophyll.

### Antioxidant Capacity

The ABTS scavenging capacity of wheatgrass was from 1.05 ± 0.10 to 2.69 ± 0.16 mg TE/g FW, shown in Figure 1J, and was rival to some herbal plants (*Leguminosae, Bignoniaceae, Moraceae, Pluchea* family, and so on) from the Amazonian region or Indonesia (78, 79) or some common fruits and vegetables, such as lemon, onion, parsley, sweet potato, and vegetable amaranths (80–82). According to the MANOVA results, the overall changing mode of ABTS bleaching ability was found decreasing from D12 to D24 and then rising on D27. Both the seedling age and abiotic treatments could significantly affect the ABTS scavenging capacity which could attribute to the enhancement of the phytochemicals in the plant. In addition, the seedling age influenced the ABTS radical scavenging ability more efficiently than the abiotic treatments [*F*_(seedlingage)_ = 22.27 > *F*_(abiotictreatment)_ = 5.63, *p* < 0.01], coinciding with the variation of functional chemical content. The treated groups that could improve the ABTS scavenging abilities were groups of NaCl 200, UV5, UV10, and UV20, discording to the previous studies that 25~100 mM NaCl reduced the ABTS scavenging ability in a short time (83). Specifically, on D21 and D27, all the groups showed a higher scavenging capacity (significantly or non-significantly) than the control.

The DPPH scavenging ability of our study ranged from 0.61 ± 0.12 to 1.47 ± 0.13 mg TE/g FW (Figure 1K), which was much higher than different vegetable amaranths (84, 85). The DPPH bleaching ability, on basis of MANOVA results, was significantly influenced by the seedling age and abiotic treatments, of which the former affected the DPPH radical scavenging ability more efficiently [*F*_(seedlingage)_ = 59.87 > *F*_(abiotictreatment)_ = 18.93, *p* < 0.01], coinciding with the variation of functional chemical content. Except for the UV10~60 treatments, all other treatments could markedly improve the DPPH bleaching ability, discording to the previous studies that 25~50 mM NaCl decreased the DPPH bleaching ability (83). The DPPH scavenging ability decreased significantly with the prolonging of the seedling age. Another notable finding was that NaCl and PEG treated groups rather than UV groups possessed significantly higher DPPH scavenging ability than the control, especially from D19 to D27 (*p* < 0.05).

The FRAP ranged from 1.23 ± 0.24 to 2.71 ± 0.26 mg TE/g FW (Figure 1L), which was comparable to common food like sweet potatoes (80) or papaya (86). Similarly, the FRAP was more significantly affected by the seedling age than abiotic treatments [*F*_(seedlingage)_ = 39.48 > *F*_(abiotictreatment)_ = 2.87, *p* < 0.01]. Interestingly, though the abiotic treatments could affect the FRAP, no statistically differences were found between the abiotic treating groups and the control, which discord with former studies that abiotic stress could significantly augment the antioxidant activity, such as FRAP (87). The discordance could be explained by the varied ability of the phytochemicals to the reducing power.

### TOPSIS Ranking Results

The closeness coefficient (R_j_) was calculated and the ranking list was presented in Table 1. Through the comprehensive ranking of the functional phytochemicals in wheatgrass, the top five highest functional phytochemicals were observed under NaCl 200 on D21 and D27, PEG 25% on D24, NaCl 200 on D24, and UV40 on D27, respectively.

**Table 1:** TOPSIS ranking results of wheatgrass phytochemicals of different seedling ages under different abiotic treatments.

**Abiotic treatments**	**D09**		**D12**		**D15**		**D19**		**D21**		**D24**		**D27**	
	**R_**j**_**	**Ranking**	**R_**j**_**	**Ranking**	**R_**j**_**	**Ranking**	**R_**j**_**	**Ranking**	**R_**j**_**	**Ranking**	**R_**j**_**	**Ranking**	**R_**j**_**	**Ranking**
NaCl 10	**0.3412**	**108**	0.4327	74	0.3720	103	0.4596	56	0.4750	44	0.4235	83	0.3898	95
NaCl 25	**0.3229**	**109**	0.4963	34	0.3826	99	0.4542	62	0.4683	48	0.4711	45	0.4887	39
NaCl 50	0.4315	78	0.4570	58	0.3881	96	0.4183	87	0.5147	23	0.4362	72	0.4705	46
NaCl 100	0.4377	71	0.5033	30	0.4085	91	0.4975	32	0.5444	16	0.4892	37	0.5509	14
NaCl 200	0.4333	73	0.4616	54	0.4136	89	0.5084	27	**0.6674**	**1**	**0.6002**	**4**	**0.6169**	**2**
PEG 5%	0.3707	104	0.4889	38	0.4162	88	0.4780	43	0.5070	28	0.4200	86	0.4853	40
PEG 10%	0.3813	100	0.4903	35	0.4022	92	0.4570	59	0.4393	69	0.5502	15	0.4494	64
PEG 15%	0.3963	93	0.5257	20	0.4204	85	0.4630	51	0.5163	22	0.5549	11	0.5531	13
PEG 20%	0.4903	36	0.5198	21	0.4621	53	0.5791	6	0.5735	8	0.5532	12	0.5684	9
PEG 25%	0.5039	29	0.5111	25	0.5310	17	0.5297	18	0.5587	10	**0.6109**	**3**	0.5739	7
UV 5	0.4320	77	0.4670	49	0.3850	98	0.3874	97	0.4322	76	0.3425	107	0.4297	79
UV 10	0.4426	67	0.4644	50	0.3497	106	**0.3077**	**111**	0.4828	41	0.4413	68	0.4966	33
UV 20	0.4581	57	0.4249	82	**0.3207**	**110**	0.3765	102	0.4449	66	0.4983	31	0.4516	63
UV 40	0.4324	75	**0.3065**	**112**	0.3562	105	0.3922	94	0.4274	81	0.5288	19	**0.5834**	**5**
UV 60	0.4291	80	0.4485	65	0.3776	101	0.4546	60	0.4091	90	0.5122	24	0.4811	42
CK	0.4222	84	0.5103	26	0.4629	52	0.4544	61	0.4696	47	0.4388	70	0.4614	55

*Rj represents the closeness coefficient. The higher the Rj value, the higher the comprehensive content of the phytochemicals. TOPSIS, technique for order preference by similarity to ideal solution. The bold values mean the top 5 highest or lowest comprehensive phytochemicals content*.

### Correlation Analysis Results

Tables 2, 3 show the results of correlation analysis. On the 9th day, only TPC and TChl correlated positively with DPPH scavenging ability, meaning that at the early vegetative stage, the polyphenol and chlorophyll were the main antioxidants in wheatgrass (*p* < 0.01 and *p* < 0.05, respectively). On the 12th day, the TPC, TFC, and TTC were significantly or extremely significantly correlated with DPPH scavenging ability and FRAP, while TTC and TPAC correlated with ABTS bleaching capacity, suggesting that the content of these compounds started to increase and play a critical role in antioxidant activity. On the 15th day, the AAC, TTC, TFC, and TPC had a high correlation-ship with FRAP, meanwhile the TChl and TPC correlated with DPPH scavenging ability. On the 19th day, the TPC and TChl were highly related to DPPH scavenging ability and FRAP and were supposed to be the main antioxidant compounds as it was on the 21st day when TFC was included. The TTC had a negative correlation with ABTS bleaching ability (*p* < 0.01) on the 19th day.

**Table 2:** Correlation coefficients of total polyphenols, total flavonoids, total proanthocyanins, total triterpenes, total soluble polysaccharides, ascorbic acid, total chlorophyll, ABTS and DPPH scavenging ability, and ferric iron-reducing antioxidant power of wheatgrass on the 9th~19th harvest day^a^^,^^b^.

**D09**	**TPC**	**TFC**	**TPAC**	**TTC**	**TSPC**	**AAC**	**TChl**	**ABTS**	**DPPH**	**FRAP**
TPC	1	0.014	0.101	0.361^*^	−0.053	0.118	0.302^*^	0.011	0.517^**^	0.038
TFC		1	0.099	−0.070	−0.140	0.084	−0.028	0.046	0.125	0.018
TPAC			1	−0.257	−0.012	−0.110	−0.109	0.106	0.190	−0.105
TTC				1	0.154	0.295^*^	0.306^*^	0.061	0.047	0.265
TSPC					1	0.027	0.300^*^	0.282	0.119	0.032
AAC						1	0.054	0.182	−0.194	0.034
TChl							1	0.100	0.311^*^	0.249
ABTS								1	−0.031	0.070
DPPH									1	0.022
FRAP										1
**D12**	**TPC**	**TFC**	**TPAC**	**TTC**	**TSPC**	**AAC**	**TChl**	**ABTS**	**DPPH**	**FRAP**
TPC	1	0.713^**^	0.081	0.438^**^	0.399^**^	−0.056	0.047	0.256	0.533^**^	0.526^**^
TFC		1	−0.156	0.267	0.163	−0.018	0.083	0.013	0.329^*^	0.416^**^
TPAC			1	0.070	0.357^*^	0.180	−0.570^**^	0.471^**^	−0.065	0.197
TTC				1	0.192	−0.213	0.247	0.469^**^	0.417^**^	0.329^*^
TSPC					1	0.243	−0.084	0.450^**^	0.255	0.552^**^
AAC						1	−0.340^*^	0.175	−0.119	0.109
TChl							1	−0.190	0.169	−0.010
ABTS								1	0.368^*^	0.294^*^
DPPH									1	0.263
FRAP										1
**D15**	**TPC**	**TFC**	**TPAC**	**TTC**	**TSPC**	**AAC**	**TChl**	**ABTS**	**DPPH**	**FRAP**
TPC	1	0.061	−0.131	0.216	0.389^**^	−0.043	0.278	−0.055	0.331^*^	0.445^**^
TFC		1	−0.169	0.474^**^	0.320^*^	0.149	0.082	0.189	0.267	0.301^*^
TPAC			1	−0.350^*^	−0.021	−0.015	−0.155	0.049	−0.199	−0.210
TTC				1	0.095	0.344^*^	0.009	0.029	0.263	0.388^**^
TSPC					1	0.444^**^	−0.183	−0.044	0.057	0.499^**^
AAC						1	−0.579^**^	0.002	−0.095	0.531^**^
TChl							1	−0.034	0.685^**^	−0.070
ABTS								1	0.009	0.063
DPPH									1	0.309^*^
FRAP										1
**D19**	**TPC**	**TFC**	**TPAC**	**TTC**	**TSPC**	**AAC**	**TChl**	**ABTS**	**DPPH**	**FRAP**
TPC	1	0.332^*^	−0.001	−0.142	0.226	−0.058	0.221	0.232	0.524^**^	0.419^**^
TFC		1	0.356^*^	−0.087	0.063	−0.106	−0.075	−0.239	0.032	0.038
TPAC			1	−0.148	0.189	0.528^**^	−0.665^**^	0.229	−0.415^**^	−0.034
TTC				1	0.119	0.200	0.196	−0.464^**^	−0.082	0.192
TSPC					1	0.479^**^	0.087	−0.043	0.230	0.321^*^
AAC						1	−0.445^**^	0.151	−0.290^*^	0.149
TChl							1	−0.406^**^	0.662^**^	0.360^*^
ABTS								1	−0.130	−0.099
DPPH									1	0.558^**^
FRAP										1

a *Correlations between the data obtained were run using a standard Pearson's correlation*.

b** *P < 0.01*;

* *P < 0.05 (two–tailed)*.

**Table 3:** Correlation coefficients of total polyphenols, total flavonoids, total proanthocyanins, total triterpenes, total soluble polysaccharides, ascorbic acid, total chlorophyll, abts and dpph scavenging ability, and ferric iron-reducing antioxidant power of wheatgrass on the 21st~27th harvest day^a^^,^^b^.

**D21**	**TPC**	**TFC**	**TPAC**	**TTC**	**TSPC**	**AAC**	**TChl**	**ABTS**	**DPPH**	**FRAP**
TPC	1	0.234	−0.016	−0.064	0.580^**^	0.617^**^	0.351^*^	0.443^**^	0.588^**^	0.331^*^
TFC		1	−0.132	−0.402^**^	−0.092	−0.014	0.596^**^	−0.242	0.538^**^	0.353^*^
TPAC			1	−0.111	0.365^*^	0.152	−0.111	0.146	−0.036	−0.077
TTC				1	−0.129	0.097	−0.390^**^	0.049	−0.169	−0.043
TSPC					1	0.599^**^	0.068	0.457^**^	0.099	0.113
AAC						1	0.120	0.123	0.104	0.266
TChl							1	−0.201	0.669^**^	0.408^**^
ABTS								1	0.179	−0.166
DPPH									1	0.517^**^
FRAP										1
**D24**	**TPC**	**TFC**	**TPAC**	**TTC**	**TSPC**	**AAC**	**TChl**	**ABTS**	**DPPH**	**FRAP**
TPC	1	0.522^**^	0.014	0.461^**^	0.517^**^	0.519^**^	−0.050	0.514^**^	0.248	0.479^**^
TFC		1	−0.035	−0.096	−0.096	−0.076	0.586^**^	0.121	0.611^**^	0.271
TPAC			1	0.042	0.034	0.012	−0.027	0.149	0.128	−0.178
TTC				1	0.734^**^	0.685^**^	−0.609^**^	0.384^**^	−0.366^*^	0.384^**^
TSPC					1	0.793^**^	−0.652^**^	0.469^**^	−0.458^**^	0.468^**^
AAC						1	−0.708^**^	0.417^**^	−0.518^**^	0.433^**^
TChl							1	−0.221	0.854^**^	−0.168
ABTS								1	0.084	0.088
DPPH									1	−0.076
FRAP										1
**D27**	**TPC**	**TFC**	**TPAC**	**TTC**	**TSPC**	**AAC**	**TChl**	**ABTS**	**DPPH**	**FRAP**
TPC	1	0.203	0.221	0.612^**^	0.741^**^	0.681^**^	−0.415^**^	0.589^**^	0.097	0.712^**^
TFC		1	−0.101	0.152	−0.177	−0.319^*^	0.498^**^	−0.079	0.492^**^	0.087
TPAC			1	0.415^**^	0.293^*^	0.303^*^	−0.403^**^	0.324^*^	−0.280	0.189
TTC				1	0.677^**^	0.549^**^	−0.378^**^	0.331^*^	−0.157	0.453^**^
TSPC					1	0.845^**^	−0.734^**^	0.542^**^	−0.345^*^	0.577^**^
AAC						1	−0.690^**^	0.516^**^	−0.367^*^	0.551^**^
TChl							1	−0.576^**^	0.555^**^	−0.343^*^
ABTS								1	0.044	0.498^**^
DPPH									1	−0.025
FRAP										1

a *Correlations between the data obtained were run using a standard Pearson's correlation*.

b** *P < 0.01*;

* *P < 0.05 (two-tailed)*.

On 24th and 27th days, it was an interesting finding that the TTC, TChl, and AAC were highly positively correlated with ABTS and FRAP scavenging ability (*p* < 0.01) but were negatively correlated with DPPH, the reason for this was unclear. TPC showed a high correlation with ABTS and FRAP, and the TFC was correlated with DPPH (*p* < 0.01).

### PCA and HHCA Reveal Differences in the Main Functional Phytochemicals

**Figure 2:**
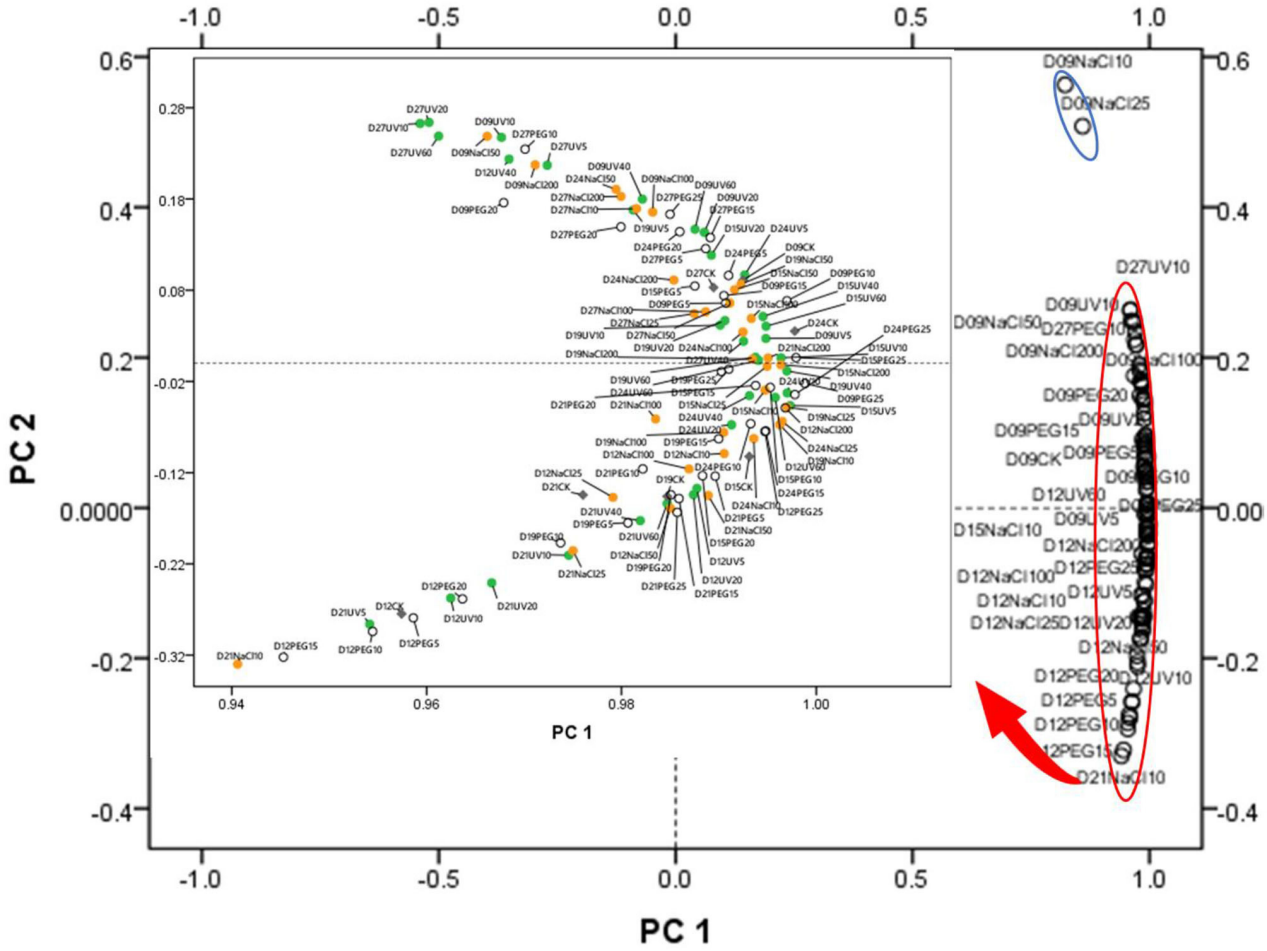
Principal component analysis (PCA) of the abiotic stress (15 kinds of treatments and one control group) × seedling ages (from D9 to D27) interactions. PC 1 explained 96.527% of the variance which correlated with total polyphenol content (TPC), total flavonoid content (TFC), total triterpene content (TTC), ABTS, DPPH, and ferric ion reducing antioxidant power (FRAP), while PC 2 took up for 2.569% of the variance and correlated with total proanthocyanins content (TPAC), total Soluble polysaccharides content (TSPC), ascorbic acid content (AAC), and total chlorophyll content (TChl). The green dots represent the UV treatments, the orange dots represent the NaCl treatments, the blank dots mean the PEG treatments, and the gray ones mean the control group (CK).

Principal component analysis and HHCA were further performed to depict the differences in the seven main functional chemicals of different abiotic treatments and antioxidant ability. The PCA plot (Figure 2) exhibited the similarities and differences among the abiotic treated groups of different seedling ages. PC 1 explained 96.527% of the variance which correlated with TPC, TFC, TTC, ABTS, DPPH, and FRAP, while PC 2 took up for 2.569% of the variance and correlated with TPAC, TSPC, AAC, and TChl. The samples were not separated from different abiotic treatments into groups, suggesting that each abiotic treatment did not have a relatively distinct phytochemical.

**Figure 3:**
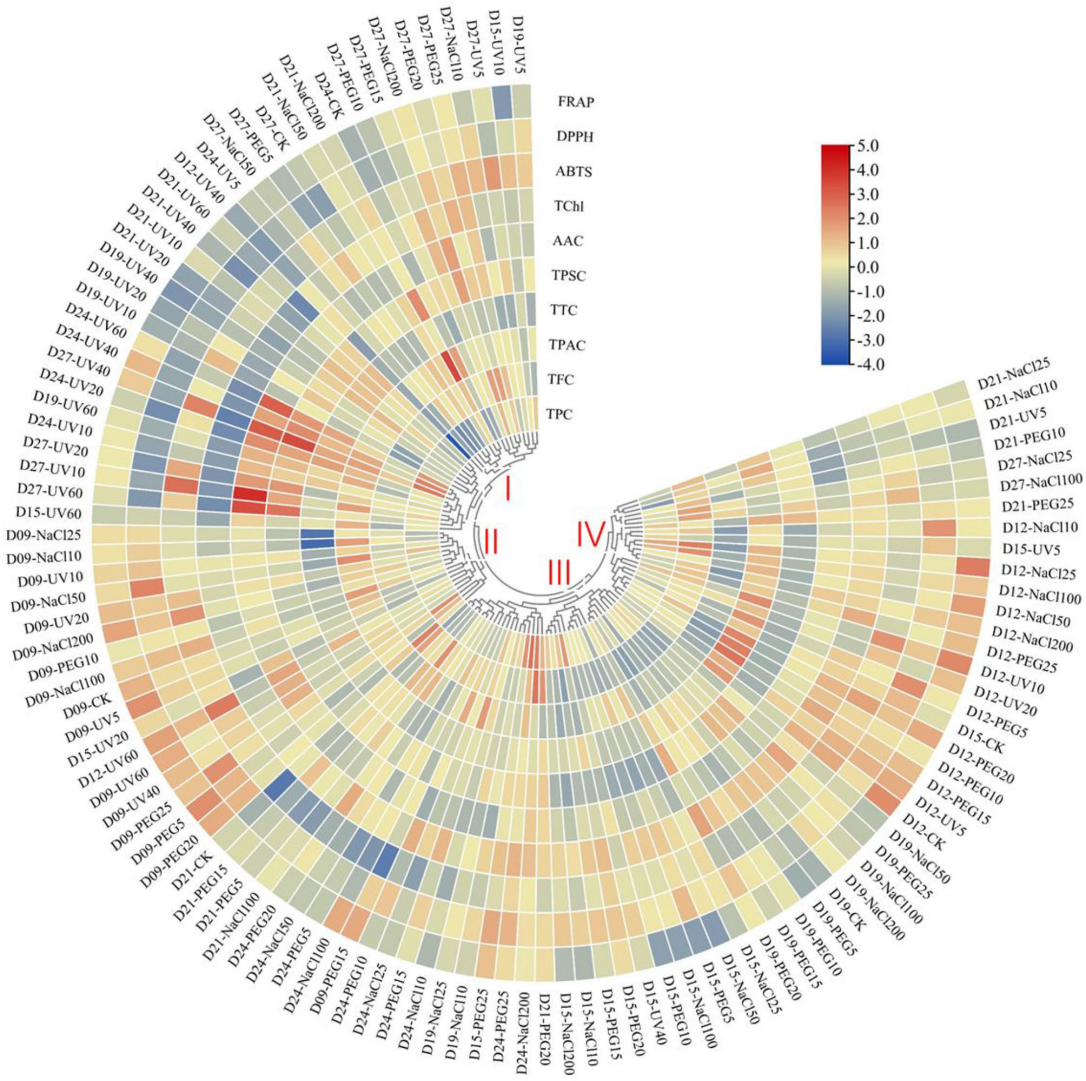
Heatmap hierarchical clustering analysis (HHCA) of the various abiotic treatments on different seedling ages of wheatgrass. Each value of the seven main functional phytochemicals and three antioxidant activity indices of abiotic treatments are visualized in a column, and each of the treatments is represented by a row. The abundance of each phytochemical content is represented by a bar with unique color. Red means upregulation, and blue means downregulation. The heatmap is constructed by TBtools software. Distance methods are using Pearson's distance.

In the present study, the HHCA demonstrated abiotic variations in terms of the relative content of phytochemicals and antioxidant capacity (Figure 3). Based on the distinct accumulation patterns, the abiotic treatments could be divided into four main clusters. The abiotic treatments in cluster I generally contributed to the decrease in the value of ABTS scavenging ability, DPPH scavenging ability, FRAP, TPC, and TChl, but increased the TSPC value. The ones in cluster II roughly contributed to the upregulation of the antioxidant capacity and TPAC. The ones in cluster III contributed to the slight upregulation of TChl and DPPH scavenging ability and slight downregulation of the value of other detected items. The abiotic treatments in cluster IV roughly contributed to the augment of antioxidant ability, TChl, TPC, and TTC value and the decrease of TPAC and TSPC.

## Conclusion

The antioxidant activity and functional phytochemical content of wheatgrass could be significantly affected by both the stress treatments and seedling ages, while the latter affected phytochemicals except chlorophyll more efficiently. The different treatments did not always lead to an increase in the content of these functional compounds supposedly but resulted in the different accumulation patterns. Through the average Rj value (data not shown), the treatments with higher comprehensive phytochemical values were PEG 15~25% and NaCl 100~200. The artificial abiotic treatments would lead to some reduction in the yield though they could somehow elevate the content of the compounds, hence, taking into consideration the cost of the treatment application, it is better to harvest the wheatgrass at a particular seedling age to acquire better nutritional value.

The highest TPC, TFC, TPAC, TTC, TSPC, AAC, and TChl were 2.89 ± 0.01 mg GAE/g FW, 4.47 ± 0.11 mg RE/g FW, 0.95 ± 0.76 mg CE/g FW, 12 ± 2.3 mg UAE/g FW, 2.41 ± 0.11 mg GE/g FW, 15.32 ± 0.05 mg/g FW, 11.6 ± 0.02 mg/g FW, respectively, and were found in groups of PEG25% on D24, NaCl 200 on D24, NaCl 200 on D21, PEG 15% on D12, UV 40 on D27, UV10 on D27, and PEG20% on D19, which could serve as a theoretical basis to high yielding of the individual functional compounds. The time-dependent pathway on how the functional compounds were regulated between different stress-tolerant cultivars was complex and still not clear; hence, it needs further investigation.

## Data Availability Statement

The original contributions presented in the study are included in the article/supplementary material, further inquiries can be directed to the corresponding author.

## Ethics Statement

The field experiments in this research were carried out as per provisions of National Standards GB/T 27476.1-2014—Safety in testing laboratories (China) and National standards GB 19489-2008—Laboratories—General requirements for biosafety (China) issued by *General Administration of Quality Supervision, Inspection and Quarantine of the People's Republic of China* and *Standardization Administration of China*.

## Author Contributions

BJ designed and funded the research. GG sponsored the publication fee and helped revise work of the manuscript. MR, YB, FG, WY, XX, MS, JW, and RC were responsible for the methodology, conducting, and data recording. LX helped in statistics handling. XZ and FF funded the research equally. QC drafted the manuscript and took the duty of data handling and revising the study. All authors contributed to the article and approved the submitted version.

## Conflict of Interest

The authors declare that the research was conducted in the absence of any commercial or financial relationships that could be construed as a potential conflict of interest.

## Publisher's Note

All claims expressed in this article are solely those of the authors and do not necessarily represent those of their affiliated organizations, or those of the publisher, the editors and the reviewers. Any product that may be evaluated in this article, or claim that may be made by its manufacturer, is not guaranteed or endorsed by the publisher.
